# Predictors of conversion to surgery in pituitary apoplexy: Insights from a Spanish multicenter observational study

**DOI:** 10.1016/j.jcte.2025.100399

**Published:** 2025-05-11

**Authors:** Betina Biagetti, Esteban Cordero Asanza, Carlos Pérez-López, Víctor Rodríguez Berrocal, Almudena Vicente, Cristina Lamas, Fernando Guerrero-Pérez, Andreu Simó-Servat, Guillermo Serra, Ana Irigaray Echarri, M. Dolores Ollero, Inmaculada González Molero, Rocío Villar-Taibo, María Dolores Moure Rodríguez, Pablo García-Feijoo, María Noelia Sánchez Ramirez, Alba Gutiérrez Hurtado, Vanessa Capristan-Díaz, Rosa Camara, Marta Gallach, Eva Safont Perez, Victoria González Rosa, Soralla Civantos-Modino, Elena Martinez-Saez, Edelmiro Menéndez Torre, Anna Aulinas, Pedro Iglesias, Juan J. Diez, Ignacio Bernabéu, Cristina Álvarez-Escolá, Manel Puig-Domingo, Marta Araujo-Castro, On behalf of neuroendocrinology Task Force of the Spanish Society of Endocrinology and Nutrition

**Affiliations:** a Endocrinology & Nutrition Department, Hospital Universitario Vall de Hebrón, CIBERER U747 (ISCIII), ENDO-ERN, Universitat Autónoma de Bracelona, Barcelona, Spain; b Neurosurgery Department, Hospital Universitario Vall d’Hebrón, Departament de Cirurgia i Ciències Morfològiques, Universitat Autònoma de Barcelona, Barcelona, Spain; c Department of Neurosurgery, Hospital Universitario La Paz, Madrid, Spain; y Universidad Europea de Madrid, Faculty of Medicine, Health and Sports Madrid, Madrid, Spain; d Department of Neurosurgery, Hospital Universitario Ramón y Cajal, Madrid, Spain; e Endocrinology & Nutrition Department, Hospital Universitario de Toledo, Toledo, Spain; f Endocrinology & Nutrition Department, Complejo Hospitalario Universitario de Albacete, Albacete, Spain; g Department of Endocrinology, Hospital Universitari de Bellvitge, L’Hospitalet de Llobregat, Barcelona, Spain; h Department of Endocrinology and Nutrition, Mutua de Terrassa University Hospital, Terrassa, Spain; i Department of Endocrinology, Son Espases University Hospital, Palma de Mallorca, Spain; j Department of Endocrinology, University Hospital of Navarre, Pamplona, Spain; k Endocrinology & Nutrition Department, Hospital Regional Universitario de Málaga, IBIMA Plataforma BIONAND Málaga, Spain; l Endocrinology & Nutrition Department, Hospital Universitario de Santiago de Compostela, Galicia, Spain; m Endocrinology & Nutrition Department, Hospital Universitario de Cruces, Bilbao, Spain; o Endocrinology & Nutrition Department, Hospital Universitario Central de Asturias, Instituto de Investigación del Principado de Asturias (ISPA), Asturias, Spain; p Department of Endocrinology, Hospital Universitario Puerta de Hierro Majadahonda, Instituto de Investigación Sanitaria Puerta de Hierro Segovia de Arana, Majadahonda, Spain; q Department of Medicine, Universidad Autónoma de Madrid, Spain; r Endocrinology & Nutrition Service, La Fe University Hospital, Valencia, Spain; s Endocrinology & Nutrition Department, Hospital de la Santa Creu i Sant Pau, IR-SANTPAU, CIBERER-U747 (ISCIII), ENDO-ERN, Barcelona, Spain; t Endocrinology & Nutrition Department, Hospital Hospital Universitario Insular de Gran Canaria, Spain; u Endocrinology & Nutrition Department, Hospital Universitario Fuenlabrada, Madrid, Spain; v Pathology Department, Hospital Universitario Vall de Hebrón, Universitat Autónoma de Barcelona, Barcelona, Spain; w Endocrinology Department, Hospital Universitario La Paz, Madrid, Spain; x Endocrinology & Nutrition Service, Germans Trias Hospital and Research Institute, Badalona, Centro de Investigación Biomédica en Red de Enfermedades Raras U747, Autonomous University of Barcelona, Barcelona, Spain; n Department of Endocrinology and Nutrition, Hospital Universitario Ramón y Cajal, Madrid, Spain

**Keywords:** Apoplexy, Pituitary, Transsphenoidal surgery, Conservative management, Conservative failure, Conversion to surgery

## Abstract

**Background:**

Pituitary apoplexy (PA) is a rare but potentially life-threatening condition. While conservative management is an option in selected cases, predictors of conversion to surgery after initial conservative management remain unclear.

**Objective:**

To identify predictors of transitioning to surgery in PA who were initially managed conservatively, and to assess the timing and impact of surgical conversion.

**Methods:**

This multicenter observational study included 134 patients with PA initially managed conservatively. Patients were categorized into successful conservative management (no surgery or surgery scheduled after 30 days) and conversion to surgery (surgery within 8–30 days). Logistic and Cox regression analyses were performed to identify predictors of conversion to surgery and time to transition, respectively.

**Results:**

Among the 134 patients enrolled, the median age was 61.4 years (interquartile range: 16.0) years and 93 (69.4 %) men], 69 (51.5 %) ultimately required surgery, with most transitions occurring within the first two weeks. In logistic regression analysis, larger tumor size (OR: 1.09, 95 % CI: 1.02–1.16) and higher BMI (OR: 1.11, 95 % CI: 1.01–1.22) were independently associated with conversion to surgery. However, Cox regression did not identify any variables predicting time to transition. Additionally, patients who converted to surgery had a significantly longer hospital stay (21.0 vs. 7.5 days, p < 0.01).

**Conclusion:**

Half of the patients initially managed conservatively required convertion to surgery. Tumor size and BMI were associated with an increased likelihood of surgery, but no factors predicted when surgery would occur, suggesting that the decision to conversion to surgery may be influenced by multiple clinical factors rather than a single determinant.

## Introduction

Acute pituitary apoplexy (PA) is a rare but potentially life-threatening endocrine and neurosurgical emergency caused by hemorrhage or infarction within the pituitary gland [1,2]. It is considered a rare condition, with an estimated incidence of 6.2 cases per 100,000 population [1,2].The condition often presents abruptly with severe headache, visual disturbances, and pituitary dysfunction, leading to an increased risk of morbidity and mortality if not promptly diagnosed and managed [3]. Notably, in over 80 % of cases, PA is the first clinical manifestation of a previously undiagnosed pituitary adenoma [4,5]. While some risk factors have been associated with PA [[5], [6], [7]], the unpredictable nature of this syndrome makes it a complex entity in clinical practice, requiring a tailored approach to treatment.

Historically, the standard of care for PA has been urgent surgical decompression, particularly in patients presenting with neuro-ophthalmological compromise [8] However, over the past decade, emerging evidence has challenged the absolute necessity of immediate surgery in all cases. Recent multicenter studies have suggested that conservative management, consisting of close monitoring, corticosteroid therapy, and supportive care, can achieve outcomes comparable to those of surgical intervention in select patient groups [[9], [10], [11]]. Despite these findings, surgical treatment remains the predominant approach in more than 60 % of PA cases, particularly in patients with larger tumors, neuro-ophthalmological deficits and with higher Pituitary Apoplexy Scores (PAS) [5,10,11].

The decision to conversion to surgery after initial conservative management is influenced by multiple factors beyond PAS, including patient comorbidities, tumor size, degree of optic chiasm compression, and response to initial medical therapy. In fact, tumor size, body mass index (BMI) and obesity-related inflammation may contribute to worse clinical outcomes [5,7]. In practice, some patients initially managed conservatively may undergo surgery not only due to persistent symptoms, such as headache or visual disturbances, but also based on patient preference or medical judgment in the absence of clear clinical deterioration. This raises an important clinical question: what distinguishes patients who remain on conservative treatment from those who ultimately conversion to surgery, and how can we better identify those at higher risk of requiring intervention?

## Patients and methods

This is a sub-study of the Apoplexy observational, retrospective Spanish nationwide registry, which collected real-world clinical data, medical practice and outcomes of patients diagnosed with acute PA between 2010 and 2023. More details on the study have been previously published [5,11].

The Spanish Society of Endocrinology and Nutrition (SEEN) endorsed the study, involving 18 medical centers across Spain. Approved by the Vall d’Hebron University Hospital ethics committee (No. PR(AG)577–2021), it adhered to the Declaration of Helsinki and good clinical practices. Patient confidentiality was safeguarded under Spanish data protection laws, and informed consent was obtained from those in active follow-up. The inclusion criteria for participating in this study were: (I) to have a non-functioning pituitary adenoma (NFPA) and (II) to continue conservative management after one week of the PA diagnosis. Functioning pituitary adenomas were excluded due to their distinct clinical course and treatment priorities that may influence management decisions.

### Definition of variables

**Fig. 1:**
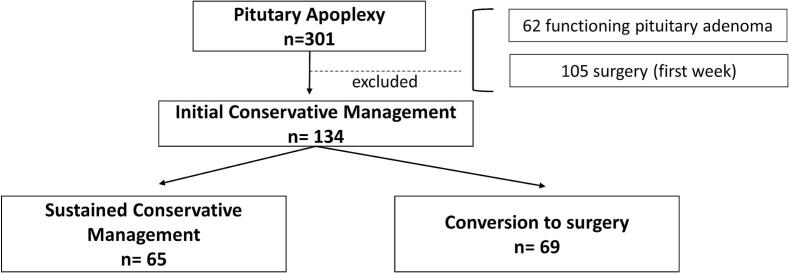
Study Flowchart: Patient Selection and Management Pathways in Pituitary Apoplexy. Fig. 1**Legend:**.Fig. 1 shows the flow diagram of initial conservative management and surgical conversion to surgery. A total of 301 patients diagnosed with pituitary apoplexy were assessed. From this group, 62 patients with functioning pituitary adenomas were excluded to maintain a homogeneous non-functioning cohort. Additionally, 105 patients who underwent early surgery (within the first 7 days of diagnosis) were excluded, as the study focused on patients who were initially managed conservatively. The final cohort consisted of 134 patients, of whom 65 remained on conservative management and 69 eventually converted to surgery between days 8 and 30.

Based on previous studies [[12], [13], [14], [15], [16]], surgical group was defined as patients who underwent an early surgery within the first week after PA diagnosis and these patients were excluded from the study. The selected window of 8–30 days reflects the typical timeframe for reassessing conservative management decisions, as described in the same studies **(**Fig. 1**)**.

The Conservative management group was established when patients did not have surgery in the first week and this group was further subdivided into two groups:

i. *Sustained conservative management*, patients who did not undergo surgery or surgery was schedule 30 days after PA.
ii. *Conversion to surgery*, patients who initiated conservative management and were operated after one week from 8 to 30 days after PA.

The decision to choose a conservative or a surgical treatment was made upon clinical judgement of the treating physician or a multidisciplinary team considering factors such as symptom severity (e.g., visual loss, ophthalmoplegia), radiological findings (tumor size, chiasmal compression), comorbidities, and the patient's overall clinical status. Patients in the conversion group underwent surgery due to lack of improvement, persistent symptoms, or based on medical judgment, even in the absence of new or worsening symptoms. All patients were discharged when considered clinically stable.

The maximum tumor diameter (D) was measured on imaging and used to classify tumors as microadenomas (<10 mm) or macroadenomas (≥10 mm). Cavernous sinus invasion was assessed using the Knosp classification [17], with Knosp scores > 2 considered indicative of invasive pituitary adenomas.

Chiasmal compression was recorded as a binary variable (yes/no), based on medical records. Extra-pituitary involvement was defined as any tumor extension beyond the sella, including chiasmal compression or cavernous sinus invasion.

The Pituitary Apoplexy Score (PAS) was calculated for each patient using Giritharan et al. criteria [18]. Comorbid conditions, including hypertension, diabetes, and dyslipidemia, were defined as active diagnoses or requiring pharmacological treatment, as documented in medical records.

Hypopituitarism was established according to current diagnostic criteria [[19], [20], [21]].

Hyponatremia was defined as a serum sodium concentration below 135 mEq/L in the first blood test after admission.

Outcome measures included conversion to surgery, the time until conversion to surgery and length of hospital stay, calculated from the date of PA diagnosis to the date of hospital discharge.

The reason for transitioning to surgery after initial conservative management was extracted from medical records. Failure was attributed to lack of symptom improvement or worsening, including persistent headache, oculomotor palsy, or visual disturbances, as well as patient preference. When none of these factors were identified, the reason for surgery was categorized as a medical decision.

### Statistical analysis

Categorical variables were reported as absolute values (n) and percentages (%), while continuous variables were expressed as medians and interquartile ranges (IQRs) calculated as Q3 minus Q1. Comparisons between the successful conservative management and conversion to surgery groups were performed using the chi-square test or Fisher's exact test for categorical variables, and the *t*-test or Mann-Whitney *U* test for continuous variables, as appropriate.

To identify predictors of conversion to surgery, logistic regression analysis was used to estimate odds ratios (OR) and 95 % confidence intervals (CIs), while Cox proportional hazards regression was performed to assess hazard ratios (HR) and 95 % CIs for time to conversion. Both univariate and multivariate models were applied, with variables selected for multivariate analysis based on clinical relevance and statistical significance in univariate analysis (p < 0.10).

The Kaplan-Meier method was used to estimate time to conversion. Hazard function analysis was conducted to evaluate variations in conversion risk over time. A p-value of < 0.05 was considered statistically significant for all comparisons.

All statistical analyses were conducted using STATA 16.0 (StataCorp LP, College Station, TX, USA).

## Results

### Baseline characteristics

Among 134 patients with PA who underwent conservative management, 65 (48.5 %) remained on conservative treatment, while 69 (51.5 %) eventually transitioned to surgery **(**Fig. 1**)**.

Table 1 shows the baseline characteristics of the patients. The median age was 61.4 years (IQR 16.0), and the majority of patients were male (69.4 %), without statistical differences between groups. The median duration of symptoms before diagnosis was similar in both groups (2 days, p = 0.16) and headache was the most common symptom (91 %), occurring at comparable rates in both groups.

**Table 1:** Baseline clinical characteristics of patients with pituitary apoplexy according to conservative treatment outcomes.

Variable	Total (N = 134)	Sustained conservative management (N = 65)	Conversion to surgery (N = 69)	P value
Gender male, n (%)	93(69.4)	44 (67.7)	49 (71.0)	0.68
Age (years), p50 (IQR)	61.4 (16.0)	63.4 (17.2)	59.6 (15.0)	0.19
Time with symptoms (days), p50 (IQR)	2 (4)	2 (3)	2 (4)	0.16
Headache, n (%)	122 (91)	60 (92.3)	62 (89.9)	0.61
Hypopituitarism, n (%)N = 119	74 (62.2)	36 (62.1)	38 (62.3)	0.98
Hyponatremia, n (%)	42 (31.6)	211 (32.8)	21(30.4)	0.77
Maximum diameter (mm), p50 (IQR)	23.5 (8.6)	21.1 (8.5)	25.6 (8.1)	<0.01
Extra pituitary involvement	105 (79.6)	46 (73.0)	59 (85.5)	0.08
Optic chiasm compression, n (%)	80 (60.6)	34 (54.0)	46 (66.7)	0.14
Knosp 3–4, n (%) n = 118	33 (28.0)	15 (26.3)	18 (29.5)	0.70
Pituitary apoplexy Score 0	35 (26.1)	21 (32.3)	14 (20.3)	0.75
1	37 (27.6)	17 (26.2)	20 (29.0)	
2	21 (15.7)	10 (15.4)	11 (15.9)	
3	17 (12.7)	8 (12.3)	9 (13.0)	
4	15 (11.2)	5 (7.7)	10 (14.5)	
5	7 (5.2)	3 (4.6)	4 (5.8)	
6	2 (1.5)	1 (1.5)	1 (1.5)	
Previously known pituitary tumor, n (%)	20 (14.9)	13 (20.0)	7 (10.1)	0.11

P50: median, IQR: interquartile range.

However, some tumor characteristics may influence the likelihood of sustained conservative treatment. Patients in the conversion to surgery group had larger tumor size, (25.6 vs. 21.1 mm; p < 0.01). and a tend toward to higher rate of extra-pituitary involvement (85.5 vs 73.0 %; p = 0.08), optic chiasm compression (66.7 % vs. 54.0 %; p = 0.14), however these last differences did not reach statistical significance. In addition, we did not find statistical differences regarding PAS, neither as categorized nor dichotomized **(**Table 1**)**.

### Co-existing characteristics

Regarding co-exiting characteristics of the patients **(**Table 2**)**, a higher BMI was more frequent in the group to conversion to surgery with a median BMI of (28.9 vs. 27.3 kg/m^2^: p = 0.05). While cardiovascular risk factors including, hypertension, dyslipidemia and type 2 diabetes were equally distributed between the groups, patients with cancer history (7.3 % vs. 18.5 %, p = 0.05) and the use of anticoagulants (8.7 % vs. 21.5 %, p = 0.03) were significantly lower in the conversion group.

**Table 2:** Co-exiting characteristics of patients with pituitary apoplexy.

**Variable**	**Total** **(N = 134)**	**Sustained conservative management (N = 65)**	**Conversion to surgery** **(N = 69)**	**P value**
BMI (Kg/m^2^), p50 (IQR)	28.1 (4.7)	27.3 (4.4)	28.9 (4.9)	0.05
Type 2 diabetes, n (%)	31 (23.1)	19 (29.2)	12(17.4)	0.10
Hypertension, n (%)	72 (53.7)	35 (53.9)	37 (53.6)	0.98
Dyslipidemia, n (%)	68 (50.8)	35 (53.9)	33(47.8)	0.49
Smoking, n (%)	25 (18.7)	11 (16.9)	14 (20.3)	0.62
Cardiovascular disease, n (%)	24 (19.1)	15 (23.1)	9 (13.0)	0.13
Anticoagulant, n (%)	20 (14.9)	14 (21.5)	6 (8.7)	0.03
Antiplatelet, n (%)	16 (12.0)	10 (15.6)	6 (8.7)	0.22
Cancer, n (%)	17 (12.7)	12 (18.5)	5 (7.3)	0.05
Dynamic test, n (%)	2 (1.5)	1 (1.5)	1 (1.5)	0.99
Hematological disease, n (%)	3 (2.2)	2 (3.1)	1 (1.5)	0.52
Dopamine agonist, n (%)	1 (0.9)	1 (1.5)	0	0.48

BMI: body mass index.

### Medical treatment

We analyzed the use of corticosteroid administration within the first 24–48 h **(**Table 3**)**. Overall, 110 (84.6 %) patients received corticosteroids, with a significantly higher proportion in the conversion to surgery group (90.8 vs. 78.5 %; p = 0.05). The most frequently used corticosteroid was dexamethasone (49.5 %), followed by hydrocortisone (42.9 %). The distribution of corticosteroid type was similar in both groups. The median daily dose of dexamethasone was 10.8 mg/day, and hydrocortisone was 150 mg/day, with no significant differences between groups.

**Table 3:** Corticosteroid use in the first 24-48hs of the apoplexy.

**Variable**	**Total** **(N = 134)**	**Sustained conservative management (N = 65)**	**Conversion to surgery** **(N = 69)**	**P value**
Corticosteroids first 24-48hs N = 130 Type of corticosteroids (N = 105) • Dexamethasone • Hydrocortisone • Other	Yes = 110 (84.6) 52 (49.5) 45(42.9) 8 (7.6)	51 (78.5) 22 (43.1) 25 (49.0) 4 (7.8)	59 (90.8) 30 (55.6) 20 (37.0) 4 (7.4)	0.05 0.43
Dexamethasone dose per day	10.8 (0.9)	10.7 (1.3)	10.8 (1.1)	0.98
Hydrocortisone dose per day	150 (220)	150 (240)	150 (220)	0.83

Percentages are based on available data (missing data in 4 patients for corticosteroid use as a binary variable [yes/no], and in 5 patients for the type of corticosteroid). Type of corticosteroid is expressed as n (%); doses are presented in mg/day as median (interquartile range).

### Main reason for transitioning to surgery and length of hospital stays

**Fig. 2:**
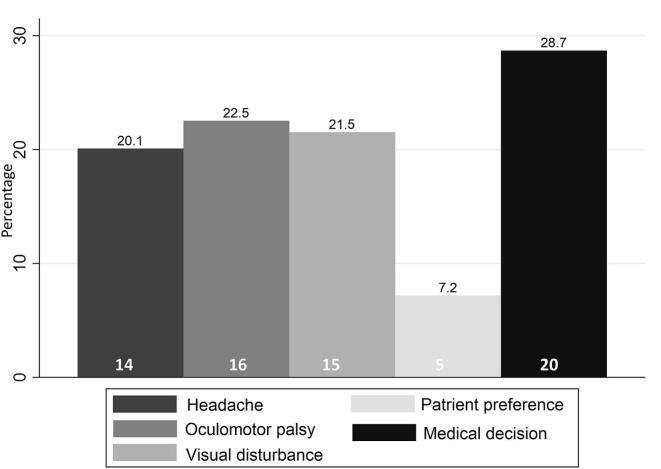
Distribution of Reasons for Conversion to Surgery in Patients with Pituitary Apoplexy. Fig. 2**Legend:** Reasons for conversion to surgery in 52 out of 69 patients with pituitary apoplexy. The most frequent cause was medical decision (28.7%), followed by oculomotor palsy (22.5%) and visual disturbances (21.1%). Medical decision” refers to surgery recommended based on clinical judgment without documented symptom worsening.

Among the 69 patients who transitioned to surgery, the reason for surgical intervention was documented in 52 cases. As shown in Fig. 2, the most frequently recorded reason was medical decision (28.7 %, n = 20), followed by oculomotor palsy (22.5 %, n = 16), visual disturbances (21.1 %, n = 15), and persistent headache (20.1 %, n = 14). Patient preference was the least common reason, accounting for 7.2 % (n = 5) of cases.

The median length of stay was significantly higher in the failure group (21.0 (10.0) vs. 7.5 (11.0) days; p < 0.01).

### Survival analysis

**Fig. 3:**
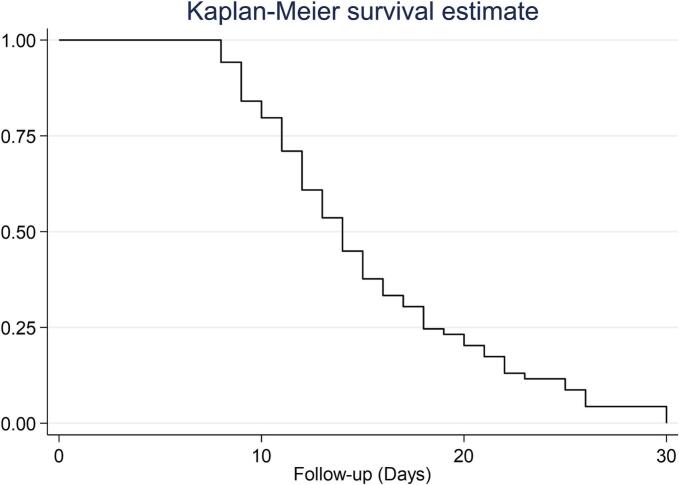
Kaplan-Meier Survival Estimate of Conversion to Surgery in Pituitary Apoplexy. Fig. 3**Legend:** The Kaplan-Meier survival curve depicts the probability of remaining on conservative management over time in patients with pituitary apoplexy.

Survival analysis in Fig. 3, provided further insight into the dynamics of conversion to surgery. By day 8, 5.8 % of patients had already transitioned, and by day 12, nearly 40 % had transitioned to surgical intervention. The median survival was 14 days. The probability of remaining on conservative treatment continued to decline sharply, with only 24.6 % of patients still managed non-surgically by day 18.

The hazard function analysis in Table 4 confirmed that the risk of conversion to surgery was not constant over time but peaked in the second week after diagnosis, especially between days 8 and 14.

**Table 4:** Kaplan-Meier Survival Analysis of Conversion to Surgery in Patients with Pituitary Apoplexy.

**Time** **days**	**At** **Risk**	**Fail**	**Lost**	**Failure function**	**[95 % CI]**
8	69	4	0	0.058	0.022–0.147
9	65	7	0	0.159	0.091–0.269
10	58	3	0	0.202	0.125–0.318
11	55	6	0	0.289	0.197–0.412
12	49	7	0	0.391	0.287–0.516
13	42	5	0	0.463	0.354–0.587
14	37	6	0	0.550	0.438–0.670
15	31	5	0	0.623	0.510–0.736
16	26	3	0	0.666	0.555–0.774
17	23	2	0	0.695	0.585–0.799
18	21	4	0	0.753	0.647–0.847
19	17	1	0	0.768	0.663–0.859
20	16	2	0	0.797	0.695–0.882
21	14	2	0	0.826	0.728–0.904
22	12	3	0	0.869	0.779–0.935
23	9	1	0	0.884	0.796–0.945
25	8	2	0	0.913	0.832–0.964
26	6	3	0	0.956	0.889–0.988
30	3	3	0	1.000	..

### Multivariate analysis

To identify predictors of conversion to surgery in PA, both logistic regression and Cox proportional hazards analysis were performed. Logistic regression was used to assess factors associated with overall conversion to surgery, while Cox regression evaluated predictors of time to conversion to surgery.

In logistic regression, larger tumor size (OR: 1.09, 95 % CI: 1.02–1.16, p < 0.01) and BMI (OR: 1.11,95 % CI:1.01–1.22, p = 0.03) remained as significant predictors of transitioning **(**Table 5**)**. Anticoagulant use and history of cancer were associated with a lower likelihood of conversion in univariate analysis but lost significance in the multivariate model.

**Table 5:** Logistic and Cox Regression Analyses for Predictors of Conservative Treatment Failure in Pituitary Apoplexy.

**Variable**	**Univariate** **OR 95 % CI**	**Multivariate** **AOR 95 % CI**	**Univariate** **HR 95 % CI**	**Multivariate** **AHR 95 % CI**
Gender male	1.17 (0.56–2.44)	0.89 (0.34–2.31)	0.84 (0.49–1.42)	0.79 (0.43–1.46)
Age	0.98 (0.96–1.01)	0.98 (0.95–1.01)	0.99 (0.98–1.01)	1.00 (0.98–1.01)
Maximum diameter	1.08 (1.02–1.12)	1.09 (1.02–1.16)	1.00 (0.99–1.03)	1.01 (0.97–1.05)
Extra pituitary involvement	2.18 (0.91–5.20)	1.13 (0.36–3.57)	0.61 (0.30–1.20)	0.58 (0.25–1.34)
BMI	1.07 (1.00–1.16)	1.11 (1.01–1.22)	0.99 (0.94–1.03)	1.00 (0.93–1.03)
Anticoagulant	0.35 (0.12–0.97)	0.37 (0.10–1.29)	1.16 (0.50–2.70)	1.31 (0.48–3.54)
Cancer	0.35 (0.11–1.04)	0.30 (0.08–1.09)	0.69 (0.27–1.74)	0.55 (0.19–1.58)
Corticosteroids first 24-48hs	2.70 (0.98–7.54)	2.95 (0.89–13.32)	1.09 (0.47–2.56)	1.24 (0.47–3.31)

In contrast, Cox regression analysis did not identify any independent predictors of conversion timing.

Multivariate models included: gender, age, tumor size, body mass index (BMI), extra-pituitary extension, anticoagulant use, cancer history, and corticosteroid use. OR: odd ratio, AOR: adjusted odd ratio.

## Discussion

This multicenter study provides real-world evidence on the limitations and challenges of conservative management in PA. Among 134 patients initially managed conservatively, more than half required surgical intervention, with most conversion to surgery occurring within two weeks after PA. Tumor size and BMI emerged as independent predictors of transitioning. In contrast, Cox regression analysis did not identify any independent predictors of conversion timing, indicating that while tumor size and BMI are associated with the overall likelihood of conversion to surgery, they do not determine when it will occur. This suggests that the decision to conversion to surgery is likely multifactorial, influenced by a combination of clinical progression, physician judgment, and individual patient characteristics rather than a single objective factor. Moreover, patients in the conversion group had a threefold longer hospital stay (21.0 vs. 7.5 days, p < 0.01), highlighting the greater healthcare burden associated with prolonged hospitalization and delayed surgical intervention. Additionally, almost one-third of surgical conversions were due to medical decision rather than clear symptom progression, suggesting that clinical judgment remains a major determinant in PA management. Similarly, the lower conversion rates observed in patients with cancer history or on anticoagulants may reflect more conservative decision-making due to perceived surgical risk.

In recent years, growing evidence suggests similar outcomes between surgical and conservative management [10,13], particularly in patients with lower PAS [[22], [23], [24], [25], [26]]. Besides, even without surgery, tumor shrinkage has been observed 3–6 months after PA [10,11], indicating that mass effect reduction may not always require immediate intervention.

In this context, our group previously studied over 300 patients with PA [5] and consistent with other reports [10,26], found that surgery remains a predominant treatment approach accounting for up to 70 % of cases. While a recent trend toward increased conservative management has been observed in the last years, from 17 % before 2017 to 30.6 % in recent years (p = 0.02)[11], a substantial proportion of patients still underwent surgery, because probably it may be the best option as per clinical judgement.

Tumor diameter was significantly larger in the conversion group, consistent with previous studies [4,10,22,26], where larger lesions were more likely to require surgery. However, while tumor size may be an important predictor, surgical decisions should not rely on this parameter alone. As noted earlier, spontaneous tumor shrinkage following PA has been well documented in conservatively managed patients [10,11] supporting the need to also consider individual factors and the potential for natural regression when deciding on surgical intervention.

Additionally, our study found that the probability of transitioning to surgery was high across all PAS categories, even in patients with PAS below 3, where a conservative approach has been proposed due to a milder clinical presentation. A similar trend was reported by Mamelak *et al.*[10] where nearly 47.8 % (32 out of 67) of patients who underwent surgery had a PAS of 0 or 1, typically considered mild cases in which surgical intervention might not be necessary.

We further confirmed this pattern in a previous study involving 215 patients with PA and NFPA, where the odds ratio (OR) for surgery in patients with PAS 1 was 3.4 (95 % CI:1.1–10.7) and remained elevated after adjusting for confounders (OR 3.5, 95 % CI: 1.0–12.1). Beyond tumor characteristics, higher BMI was significantly associated with conversion to surgery, suggesting a potential role of metabolic factors in disease progression. In this regard, metabolic risk factors [5] and obesity [7] have been linked to PA. Some types of obesity [27,28] are known to be associated with chronic low-grade inflammation, altered hemodynamics, higher intracranial pressure and prothrombotic states, which could contribute to worse outcomes in patients with PA.

While hemodynamic support, renal function, electrolytes monitoring and hormonal assessment are the standard of care in PA [2,3,[29], [30], [31]] there is no universally standardized protocol of urgent high glucocorticoid doses administration [29]. Practice varies across centers; many administer empirical corticosteroids to all patients with suspected PA due to the potential for life-threatening adrenal insufficiency, while others reserve it for cases with hemodynamic instability, altered consciousness, or visual compromise as these symptoms may indicate acute adrenal insufficiency. Our study revealed that corticosteroid therapy was not universally administered, with only 84.6 % receiving treatment within the first 24–48 h. This indicates that a substantial proportion lacked treatment, the treatment was contraindicated, or it was not registered. Although glucocorticoids prevent adrenal insufficiency, their anti-inflammatory and antiedematous effects remain uncertain [32]. Hydrocortisone is the preferred corticosteroid, though dosing relies on expert opinion rather than clinical evidence [32]. The use of high-dose dexamethasone (up to 16 mg/day) as an alternative to stress-dose hydrocortisone has not been formally studied but has been reported, particularly for its antiedematous effects [4]. Our study found no differences in corticosteroid type between groups, highlighting the need for further research to determine their impact on treatment success, optimal dosing, and role in PA management.

One of the most striking findings of our study was that the primary reason for surgery in almost one-third of cases was a medical decision not related to headache persistence, symptom progression, or patient preference. This merits further investigation, as it suggests that other factors may influence surgical decision-making in PA management.

The Kaplan-Meier survival analysis revealed that the median survival time on conservative management was 14 days, with a sharp decline in survival probability within the first two weeks. This peak likely reflects clinical reassessment windows, worsening symptoms, or failure to improve under conservative management. This timeframe may represent a critical window for reassessing conservative strategies.

Additionally, patients in the conversion to surgery group experienced significantly longer hospital stays (21.0 vs. 7.5 days, p < 0.01), averaging nearly three weeks compared to approximately one week for those who remained in conservative management. This prolonged hospitalization not only increases healthcare costs but also reflects a more complex clinical course, potentially due to delayed symptom resolution, additional interventions, or prolonged monitoring before and after surgery. These findings suggest that earlier identification of patients at risk for transitioning to surgery could improve patient selection, reduce hospital burden, and optimize resource allocation. Given the added costs and longer recovery associated with delayed surgery, the decision to proceed with surgical intervention in patients without symptom progression should be carefully considered, taking into account spontaneous tumor volume reduction, overall costs, and healthcare resource utilization in PA management. Postoperative length of stay could not be analyzed due to inconsistent documentation of surgery dates across centers.

Our study's strengths include its multicenter design, providing real-world data on conservative management failure in PA. However, its retrospective nature, the variability in clinical decision-making across centers and the lack of ethnic perspective may limit generalizability. In addition, the number of variables included in the multivariate model slightly exceeded the recommended events-per-variable ratio, which may affect model robustness and generalizability. Finally, as this study focused exclusively on NFPAs, future studies are needed to determine whether these predictors apply to functioning tumors.

Future research should focus on developing validated risk stratification models that integrate tumor characteristics, metabolic factors, initial clinical presentation, and identification of early markers of treatment response to improve surgical decision-making. Understanding which patients are most likely to fail conservative treatment could lead to more personalized management strategies and reduce unnecessary delays in surgery and prolonged hospitalization. Another important area to explore is the role of corticosteroid therapy in PA management. Given the variability in corticosteroid use and the lack of consensus on optimal dosing and choice of agent, prospective trials should investigate whether high-dose dexamethasone offers advantages over stress-dose hydrocortisone and whether corticosteroid treatment influences long-term outcomes.

Additionally, the study highlights the need to examine the factors influencing surgical decision-making, as a considerable proportion of surgeries were performed based on medical judgment rather than clear clinical deterioration. Identifying the reasons behind these decisions could help establish more standardized criteria for selecting surgical candidates and reduce healthcare burden associated with prolonged hospitalization.

## Conclusion

This study highlights that half of the patients initially managed conservatively required surgery within the first month, with most surgery occurring within two weeks. Larger tumor size and higher BMI were associated with an increased likelihood of conversion to surgery, yet no factor predicted the conversion timing. These findings suggest that while tumor burden influences the need for surgery, clinical evolution is unpredictable. A considerable proportion of surgeries were performed based on medical judgment different than clear clinical deterioration. Given the longer hospital stays associated with failed conservative management, the decision to proceed with surgical intervention in patients without symptom progression should be carefully considered, taking into account spontaneous tumor volume reduction, overall costs, and healthcare resource utilization in PA management.

## Institutional Review Board Statement

The study was conducted according to the guidelines of the Declaration of Helsinki and approved by the Ethics Committee of the Vall d’Hebron University Hospital (No. PR(AG)577-2021).

## Informed consent statement

Patient consent was waived due to the retrospective nature of the study. Only for patients who continued follow-up the informed consent was requested.

## CRediT authorship contribution statement

**Betina Biagetti:** Writing – review & editing, Writing – original draft, Methodology, Formal analysis, Data curation, Conceptualization. **Esteban Cordero Asanza:** Writing – review & editing. **Carlos Pérez-López:** Visualization. **Víctor Rodríguez Berrocal:** Visualization. **Almudena Vicente:** Visualization. **Cristina Lamas:** Visualization. **Fernando Guerrero-Pérez:** Visualization. **Andreu Simó-Servat:** Visualization. **Guillermo Serra:** Visualization. **Ana Irigaray Echarri:** Visualization. **MDolores Ollero:** Visualization. **Inmaculada González Molero:** Visualization. **Rocío Villar-Taibo:** Visualization. **María Dolores Moure Rodríguez:** Visualization. **Pablo García-Feijoo:** Visualization. **María Noelia Sánchez Ramirez:** Visualization. **Alba Gutiérrez Hurtado:** Visualization. **Vanessa Capristan-Díaz:** Visualization. **Rosa Camara:** Visualization. **Marta Gallach:** Visualization. **Eva Safont Perez:** Visualization. **Victoria González Rosa:** Visualization. **Soralla Civantos-Modino:** Visualization. **Elena Martinez-Saez:** Visualization. **Edelmiro Menéndez Torre:** Visualization. **Anna Aulinas:** Writing – review & editing, Visualization. **Pedro Iglesias:** Writing – review & editing, Visualization. **Juan J. Diez:** Writing – review & editing, Visualization. **Ignacio Bernabéu:** Visualization. **Cristina Álvarez-Escolá:** Visualization. **Manel Puig-Domingo:** Writing – review & editing, Visualization, Supervision. **Marta Araujo-Castro:** Writing – review & editing, Visualization, Supervision.

## Declaration of Generative AI and AI-assisted technologies in the writing process

During the preparation of this work the author(s) used Chat-GPT to assist with aspects of writing such as clarity, fluency, and consistency. After using this tool/service, the author(s) reviewed and edited the content as needed and take(s) full responsibility for the content of the publication.

## Funding

No funding was received for this study.

## Declaration of competing interest

The authors declare that they have no known competing financial interests or personal relationships that could have appeared to influence the work reported in this paper.

## References

[1] Briet C., Salenave S., Bonneville J.-F., Laws E.R., Chanson P. (2015). Pituitary Apoplexy. Endocr Rev.

[2] Iglesias P. (2024). Pituitary Apoplexy: An Updated Review. J Clin Med.

[3] Briet C., Salenave S., Chanson P. (2015). Pituitary apoplexy. Endocrinol Metab Clin North Am.

[4] Bujawansa S., Thondam S.K., Steele C., Cuthbertson D.J., Gilkes C.E., Noonan C. (2014). Presentation, management and outcomes in acute pituitary apoplexy: a large single-centre experience from the United Kingdom. Clin Endocrinol (Oxf).

[5] Biagetti B., Cordero Asanza E., Pérez-López C., Araujo-Castro M., Camara R., Guerrero-Pérez F. (2024). Pituitary Apoplexy: Comorbidities, Management and Outcomes. A Spanish Observational Multicenter Study. J Clin Endocrinol Metab.

[6] Ciavarra B., McIntyre T., Kole M.J., Li W., Yao W., Guttenberg K.B. (2023). Antiplatelet and anticoagulation therapy and the risk of pituitary apoplexy in pituitary adenoma patients. Pituitary.

[7] Garcia-Feijoo P., Perez Lopez C., Paredes I., Acitores Cancela A., Alvarez-Escola C., Calatayud M. (2024). Exploring risk factors of severe pituitary apoplexy: Insights from a multicenter study of 71 cases. Endocrine.

[8] Bonicki W., Kasperlik-Załuska A., Koszewski W., Zgliczyński W., Wisławski J. (1993). Pituitary apoplexy: endocrine, surgical and oncological emergency. Incidence, clinical course and treatment with reference to 799 cases of pituitary adenomas. Acta Neurochir (Wien).

[9] Almeida J.P., Sanchez M.M., Karekezi C., Warsi N., Fernández-Gajardo R., Panwar J. (2019). Pituitary Apoplexy: Results of Surgical and Conservative Management Clinical Series and Review of the Literature. World Neurosurg.

[10] Mamelak A.N., Little A.S., Gardner P.A., Almeida J.P., Recinos P., Soni P. (2024). A Prospective, Multicenter, Observational Study of Surgical vs Nonsurgical Management for Pituitary Apoplexy. J Clin Endocrinol Metab.

[11] Biagetti B., Cordero Asanza E., García-Feijoo P., Araujo-Castro M., Rodríguez Berrocal V., Serra G. (2024). Trends and Outcomes in Pituitary Apoplexy Management: A Spanish Observational Multicenter Study. Neurosurgery.

[12] Tu M., Lu Q., Zhu P., Zheng W. (2016). Surgical versus non-surgical treatment for pituitary apoplexy: A systematic review and meta-analysis. J Neurol Sci.

[13] Goshtasbi K., Abiri A., Sahyouni R., Mahboubi H., Raefsky S., Kuan E.C. (2019). Visual and Endocrine Recovery Following Conservative and Surgical Treatment of Pituitary Apoplexy: A Meta-Analysis. World Neurosurg.

[14] Sahyouni R., Goshtasbi K., Choi E., Mahboubi H., Le R., Khahera A.S. (2019). Vision Outcomes in Early versus Late Surgical Intervention of Pituitary Apoplexy: Meta-Analysis. World Neurosurg.

[15] Cabuk B., Kaya N.S., Polat C., Geyik A.M., Icli D., Anik I. (2021). Outcome in pituitary apoplexy patients, stratified by delay between symptom appearance and surgery: A single center retrospective analysis. Clin Neurol Neurosurg.

[16] Kelly P.D., Fernando S.J., Malenke J.A., Chandra R.K., Turner J.H., Chambless L.B. (2021). The Effect of Timing of Surgery in Pituitary Apoplexy on Continuously Valued Visual Acuity. J Neurol Surg B Skull Base.

[17] Knosp E, Steiner E, Kitz K, Matula C. Pituitary adenomas with invasion of the cavernous sinus space: a magnetic resonance imaging classification compared with surgical findings. Neurosurgery 1993;33:610–7; discussion 617-618. https://doi.org/10.1227/00006123-199310000-00008. 10.1227/00006123-199310000-00008 8232800

[18] Giritharan S., Gnanalingham K., Kearney T. (2016). Pituitary apoplexy - bespoke patient management allows good clinical outcome. Clin Endocrinol (Oxf).

[19] Fleseriu M., Hashim I.A., Karavitaki N., Melmed S., Murad M.H., Salvatori R. (2016). Hormonal Replacement in Hypopituitarism in Adults: An Endocrine Society Clinical Practice Guideline. J Clin Endocrinol Metab.

[20] Fleseriu M., Christ-Crain M., Langlois F., Gadelha M., Melmed S. (2024). Hypopituitarism Lancet.

[21] Iglesias P. (2024). An Update on Advances in Hypopituitarism: Etiology, Diagnosis, and Current Management. J Clin Med.

[22] Marx C., Rabilloud M., Borson Chazot F., Tilikete C., Jouanneau E., Raverot G. (2021). A key role for conservative treatment in the management of pituitary apoplexy. Endocrine.

[23] Leyer C., Castinetti F., Morange I., Gueydan M., Oliver C., Conte-Devolx B. (2011). A conservative management is preferable in milder forms of pituitary tumor apoplexy. J Endocrinol Invest.

[24] Salle H., Cane M., Rocher M., Auditeau E., Teissier M.-P., Raverot G. (2023). Pituitary apoplexy score, toward standardized decision-making: a descriptive study. Pituitary.

[25] Seo Y., Kim Y.H., Dho Y.-S., Kim J.H., Kim J.W., Park C.-K. (2018). The Outcomes of Pituitary Apoplexy with Conservative Treatment: Experiences at a Single Institution. World Neurosurg.

[26] Singh T.D., Valizadeh N., Meyer F.B., Atkinson J.L.D., Erickson D., Rabinstein A.A. (2015). Management and outcomes of pituitary apoplexy. J Neurosurg.

[27] Preda A., Carbone F., Tirandi A., Montecucco F., Liberale L. (2023). Obesity phenotypes and cardiovascular risk: From pathophysiology to clinical management. Rev Endocr Metab Disord.

[28] Hildebrandt X., Ibrahim M., Peltzer N. (2023). Cell death and inflammation during obesity: “Know my methods, WAT(son).”. Cell Death Differ.

[29] Baldeweg S.E., Vanderpump M., Drake W., Reddy N., Markey A., Plant G.T. (2016). Society for endocrinology endocrine emergency guidance: Emergency management of pituitary apoplexy in adult patients. Endocr Connect.

[30] Hannoush ZC, Weiss RE. Pituitary Apoplexy. In: Feingold KR, Anawalt B, Boyce A, Chrousos G, de Herder WW, Dhatariya K, et al., editors. Endotext, South Dartmouth (MA): MDText.com, Inc.; 2000. 25905348

[31] Rajasekaran S., Vanderpump M., Baldeweg S., Drake W., Reddy N., Lanyon M. (2011). UK guidelines for the management of pituitary apoplexy. Clin Endocrinol (Oxf).

[32] Capatina C., Inder W., Karavitaki N., Wass J.A.H. (2015). MANAGEMENT OF ENDOCRINE DISEASE: Pituitary tumour apoplexy. Eur J Endocrinol.

